# Overcoming co-product inhibition in the nicotinamide independent asymmetric bioreduction of activated C=C-bonds using flavin-dependent ene-reductases

**DOI:** 10.1002/bit.24981

**Published:** 2013-07-10

**Authors:** Christoph K Winkler, Dorina Clay, Esta van Heerden, Kurt Faber

**Affiliations:** 1 Department of Chemistry, Organic and Bioorganic Chemistry, University of GrazHeinrichstrasse 28, A-8010, Graz, Austria; 2 Department of Microbial, Biochemical and Food Biotechnology, University of the Free State9300, Bloemfontein, South Africa

**Keywords:** old yellow enzyme, ene-reductase, disproportionation, in situ co-product removal

## Abstract

Eleven flavoproteins from the old yellow enzyme family were found to catalyze the disproportionation (“dismutation”) of conjugated enones. Incomplete conversions, which were attributed to enzyme inhibition by the co-product phenol could be circumvented via in situ co-product removal by scavenging the phenol using the polymeric adsorbent MP-carbonate. The optimized system allowed to reduce an alkene activated by ester groups in a “coupled-substrate” approach via nicotinamide-free hydrogen transfer with >90% conversion and complete stereoselectivity.

## Introduction

**Figure 1:**
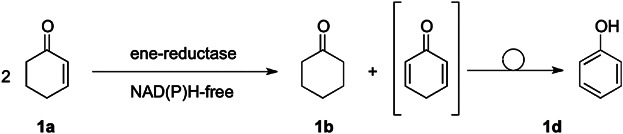
Ene-reductase catalyzed disproportionation of cyclohex-2-enone (1a).

Ene-reductases from the “old yellow enzyme” family (OYE), which catalyze the asymmetric *trans*-reduction of alkenes bearing an electron-withdrawing activating group (Stuermer et al., 2007; Toogood et al., 2010; Winkler et al., 2012) became important biocatalysts over the last few years. In the classic approach, the reduced flavin is recycled via a nicotinamide cofactor at the expense of a sacrificial hydrogen-donor cosubstrate, such as glucose, glucose-6-phosphate, formate, 2-propanol, or phosphite (Hollmann et al., 2010). Overall, this so-called “enzyme-coupled” process depends on two enzymes and two cofactors (Faber, 2011; Matsuda et al., 2009; Tauber et al., 2011; Wandrey, 2004). Several attempts were made to reduce the complexity of these systems by cancelling NAD(P)H and its recycling from the system. Direct reduction of the active site flavin was accomplished by an additional flavin-catalyst which in turn was regenerated in a light-mediated reaction by an auxiliary substrate (Grau et al., 2009; Taglieber et al., 2008). Only recently, ene-reductases were successfully employed with molar equivalents of synthetic nicotinamide mimics instead of “natural” NAD(P)H (Paul et al., 2013). We have recently proposed a nicotinamide-independent recycling system for reduced flavins based on the disproportionation (dismutation) of enones (Stueckler et al., 2010), which has been observed as catalytic promiscuity of OYEs (Fig. 1) (Buckman and Miller, 1998; Karplus et al., 1995; Vaz et al., 1995). During this reaction, an equivalent of [2H] is transferred by a single flavoprotein between two enone substrates (**1a**) yielding an oxidized (**1d**) and reduced product (**1b**) in equimolar amounts. The reductive half-reaction proceeds via the desaturation of enone **1a** (Vaz et al., 1995) forming FMNH_2_ and cyclohexa-1,4-dienone, which irreversibly tautomerises to phenol (**1d**), thereby providing a strong driving force for the overall process. The reduced flavin subsequently reduces the second equivalent of enone **1a**, which resembles the oxidative half-reaction, and closes the catalytic cycle.

**Figure 2:**
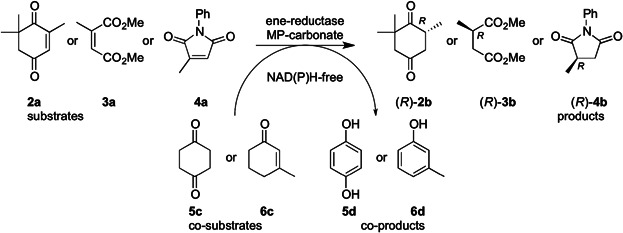
NAD(P)H-independent bioreduction of alkenes 2a–4a at the expense of H-donors 5c or 6c in presence of MP-carbonate as phenol-scavenger.

The crosswise diproportionation between two identical enone substrates (**1a**) could be turned into a directed hydrogen-transfer system by combining two different enone substrates, each serving as distinct hydrogen donor and acceptor, respectively (Fig. 2). Although the proof of principle was shown, the system was practically not applicable due to incomplete conversions (max. ≤65%). The latter were attributed to inhibition exerted by the co-product phenol. Electron-rich phenols act as strong inhibitors of OYEs through formation of stable charge-transfer complexes with the electron-deficient flavin in the active site (Abramovitz and Massey, 1976a, 1976b; Buckman and Miller, 1998; Matthews et al., 1975; Spiegelhauer et al., 2010; Stewart and Massey, 1985; Strassner et al., 1999). Prompted by the fact, that complex formation is reversible (Buckman and Miller, 1998), we aimed to overcome inhibition by reaction optimization (pH and temperature) and co-product scavenging using a solid-phase organic resin.

## Materials and Methods

### General

GC-FID analyses were carried out on a Varian 3800 using H_2_ as carrier gas (14.5 psi). HPLC analyses were performed by using a Shimadzu system equipped with a Chiracel OD-H column (25 × 0.46 cm).

Cyclohex-2-enone (**1a**), cyclohexanone (**1b**), phenol (**1d**), 4-ketoisophorone (**2a**), *N*-phenyl-2-methylmaleimide (**4a**), 1,4-dihydroxybenzene (**5d**), 3-methylcyclohex-2-enone (**6c**), and 3-methylphenol (**6d**) were purchased from Sigma-Aldrich (St. Louis, MO), 1,4-cyclohexanedione (**5c**) was from Fluka. *rac*-2,3-Epoxy-1-cyclohexanone (**1e**) (Mueller et al., 2009), dimethyl citraconate (**3a**), *rac*-dimethyl 2-methylsuccinate (*rac*-**3b**) (Stueckler et al., 2007), and *rac*-*N*-phenyl-2-methylsuccinimide (*rac*-**4b**) (Hall et al., 2007) were synthesized as previously reported. Levodione (*rac*-**2b**) was kindly provided by BASF-SE (Ludwigshafen). MP-Carbonate (loading capacity 2.5 mmol/g, mean bead size 655 µm, bead size distribution 350–1,250 µm) was obtained from Biotage.

### Source of Enzymes

12-Oxophytodienoate reductase isoenzymes OPR1 and OPR3 from *Lycopersicon esculentum* and the OYE homologue YqjM from *Bacillus subtilis* were overexpressed and purified as reported (Breithaupt et al., 2006; Hall et al., 2007; Kitzing et al., 2005). The cloning, purification, and characterization of OYE isoenzymes from yeast (OYE1 from *Saccharomyces pastorianus*, OYE2 and OYE3 from *Saccharomyces cerevisiae*) and nicotinamide-dependent cyclohexenone reductase (NCR) from *Zymomonas mobilis* were performed according to literature methods (Hall et al., 2008; Muller et al., 2007). Xenobiotic reductases XenA and XenB from *Pseudomonas putida* and *Pseudomonas fluorescens*, respectively, glycerol trinitrate reductase NerA from *Agrobacterium radiobacter*, *Kluyveromyces lactis* yellow enzyme 1 (KYE1), *Yersinia bercovieri* ene-reductase (YersER) and nitroreductase from *Salmonella typhimurium* (NRSal) were obtained as described (Durchschein et al., 2010; Yanto et al., 2010a, 2010b, 2011). *N*-Ethylmaleimide reductase (NemR) from *Escherichia coli*, pentaerythritol tetranitrate reductase (PETNr) from *Enterobacter cloacae* PB2, morphinone reductase (MR) from *P. putida* M10 and estrogen binding protein EBP1 from *Candida albicans* were obtained as recently published (Durchschein et al., 2010; Mueller et al., 2010; Winkler et al., 2013; Yanto et al., 2011). *Bacillus subtilis* YcnD and YhdA and *S. cerevisiae* Lot6p were expressed and purified as recently reported (Deller et al., 2006; Morokutti et al., 2005; Mueller et al., 2009; Sollner et al., 2007). The cloning and characterization of *Gk*OYE from *Geobacillus kaustophilus* DSM 7263 (Schittmayer et al., 2010) and the production of CrS (Opperman et al., 2008, 2010) were performed as reported.

### General Procedure A for Aerobic Enzymatic Disproportionation of Cyclohex-2-Enone (**1a**)

An aliquot of isolated enzyme (OYE1-3, CrS, EBP1, NCR, XenA, XenB, YqjM, OPR1, OPR3, NerA, *Gk*OYE, NemR, PETNr, YcnD, Lot6P, YhdA, KYE1, MR, YersER, and NRSal; protein purity >90%, protein content in reaction 100 µg/mL) was added to a microcentrifuge tube (1.5 mL) containing buffer solution (0.8 mL, 50 mM, Tris–HCl buffer; pH 7.5) and cyclohex-2-enone (**1a**, 20 mM). The mixture was agitated for 24 h at 30°C and 120 rpm using an Infors Unitron shaker and products were extracted with ethyl acetate (0.7 mL). The organic phase was dried over Na_2_SO_4_ and analyzed on GC to determine the conversion. For every test, a control was performed in the absence of enzyme.

### General Procedure B for Anaerobic Enzymatic Disproportionation of Cyclohex-2-Enone (**1a**)

An aliquot of isolated enzyme (OYE1, OYE2, CrS, EBP1, NCR, XenA, and YqjM; protein purity >90%, protein content in reaction 100 µg/mL) was added to a screw-top glass vial (2 mL) containing a degassed buffer solution (0.8 mL, 50 mM, Tris–HCl buffer; pH 7.5), cyclohex-2-enone (**1a**, 20 mM) and (optionally) MP-carbonate (up to 100 mg, 40 eq. loading capacity). The vial was flushed with argon and sealed using a screw cap lined with a teflon septum. The mixture was shaken for 24 h at 30°C and 120 rpm using an Infors Unitron shaker and products were extracted with ethyl acetate (0.7 mL). The organic phase was dried over Na_2_SO_4_ and analyzed on GC to determine the conversion. For every test, a control was performed in the absence of enzyme.

### Optimization of Reaction Conditions: Buffer-Type and pH, Reaction Time and Temperature

The optimization of reaction conditions was carried out by individual variation of every single parameter of general procedure A. For the optimization of the reaction temperature, the microcentrifuge tubes were shaken at 500 rpm in an Eppendorf thermomixer.

### General Procedure C for Anaerobic NAD(P)H-Independent Asymmetric Bioreduction of Activated Alkenes

An aliquot of isolated enzyme (OYE1-2, CrS, EBP1, NCR, XenA, YqjM, NerA, and GkOYE; protein purity >90%, protein content in reaction 100 µg/mL) was added to a screw-top glass vial (2 mL) containing a degassed buffer solution (0.8 mL, 50 mM, Tris–HCl buffer; pH 7.5), the substrate (**2a**, **3a** or **4a**, 10 mM), the H-donor (**5c** or **6c**; 10 mM) and (optionally) MP-carbonate (up to 100 mg, 40 eq. loading capacity). The vial was flushed with argon and sealed using a screw cap lined with a teflon septum. The mixture was shaken for 24 h at 30°C and 120 rpm using an Infors Unitron shaker and products were extracted with ethyl acetate (2 × 0.7 mL). The combined organic phase was dried over Na_2_SO_4_ and analyzed on GC to determine the conversion and stereoselectivity. For every test, a control was performed in the absence of enzyme. For the determination of conversion a calibration curve was established for a range of substrate/H-donor ratios in presence of MP-carbonate to compensate for the different adsorption of substrate and H-donor onto the carrier.

## Analytical Methods

### Determination of Conversion

Conversions were determined by GC-FID using a J&W HP-5 5% phenylmethylpolysiloxane capillary column (30 m × 0.32 mm, 0.25 µm film). Injector and detector temperature 300°C, split ratio 20:1. Temperature program: 100°C hold 0.5 min, 10°C/min to 240°C. Retention times: **1a**: 2.87; **1b**: 2.69; **1d**: 3.03; **1e**: 3.19; **2a**: 4.54; **2b**: 4.72; **3a**: 4.00; **3b**: 3.63; **4a**: 10.34; **4b**: 11.14; **5c**: 3.87; **5d**: 4.61; **6c**: 3.68; **6d**: 3.76 min.

### Determination of Absolute Configuration and Enantiomeric Excess

The enantiomeric excess of **2b** was determined using a β-cyclodextrin capillary column (CP Chirasil-DEX CB, 25 m × 0.32 mm, 0.25 µm film). Detector temperature 200°C, injector temperature 180°C, split ratio 25:1. Temperature program for **2b**: 90°C hold 2 min, 4°C/min to 115°C, 20°C/min to 180°C, hold 2 min. Retention times: (*R*)-**2b** 6.42, (*S*)-**2b** 6.74 min (Hall et al., 2007; Stueckler et al., 2010). The enantiomeric excess of **3b** was determined using a modified β-cyclodextrin capillary column (Chiraldex B-TA, 40 m × 0.25 mm, 0.12 µm film). Detector temperature 200°C, injector temperature 180°C, split ratio 25:1. Temperature program for **3b**: 90°C hold 4 min, 2°C/min to 110°C, 30°C/min to 180°C, hold 4 min. Retention times: (*S*)-**3b** 14.55 and (*R*)-**3b** 14.65 min (Stueckler et al., 2007, 2010). The enantiomeric excess of **4b** was determined by HPLC using *n*-heptane/i-PrOH 95:5 (isocratic) using a Chiracel OD-H column (25 × 0.46 cm) at 18°C and 1 mL/min. Retention times: (*R*)-**4b** 25.10 min; (*S*)-**4b** 29.15 min. The absolute configuration was determined as previously reported (Hall et al., 2007; Stueckler et al., 2010).

## Results and Discussion

The disproportionation-activity of a series of 22 ene-reductases was evaluated in a screening using cyclohex-2-enone (**1a**) as substrate (Fig. 1). During the course of these tests under standard conditions (pH 7.5, aerobic), the list of previously reported candidate enzymes—OYE1 from *S. pastorianus*, OYE2 and OYE3 from *S. cerevisiae*, NCR from *Z. mobilis*, EBP1 from *C. albicans* and YqjM from *B. subtilis*—could be considerably expanded by several OYE-homologs, such as NerA from *A. radiobacter* (Durchschein et al., 2010), and the thermostable OYE-variants CrS from *Thermus scotoductus* SA-01, (Opperman et al., 2008) and *Gk*OYE from *G. kaustophilus* DSM 7263 (Schittmayer et al., 2010), which were recently discovered (Table 1). Most remarkably, CrS from *T. scotoductus* SA-01 was highly active showing 55% conversion. The high dismutase-activity of OYE1, OYE2, and EBP1 was confirmed by conversions of up to 61% (Buckman and Miller, 1998; Stueckler et al., 2010; Vaz et al., 1995). Modest conversions were found using *Gk*OYE and NerA (10%), all other tested enzymes showed only low activities (<7% conversion) (Table 1, column A). An attempt to correlate the disproportionation activity with sequence-data with emphasis on the residues involved in FMN-binding, thereby modulating its redox potential, and the catalytic residues responsible for substrate binding and H^+^-donation did not reveal any apparent patterns (Table SI).

**Table 1:** Aerobic and anaerobic enzymatic disproportionation of cyclohex-2-enone (1a)

Enzyme^a^	Aerobic			Anaerobic^b^	
	pH 7.5	pH 9		pH 7.5	pH 9
Column	A	B		C	D
	c. (%)	c. (%)	epox. (%)	c. (%)	c. (%)
OYE1	58 ± 7	63 ± 6	<1	63 ± 10	64 ± 8
OYE2	61 ± 7	66 ± 7	<1	67 ± 8	65 ± 6
OYE3	7 ± 2	8 ± 1	<1	n.d.	n.d.
CrS	55 ± 4	56 ± 8	5 ± 3	62 ± 6	60 ± 6
EBP1	22 ± 7	28 ± 6	<1	25 ± 6	26 ± 6
NCR	6 ± 1	20 ± 2	<1	8 ± 1	17 ± 7
XenA	5 ± 1	8 ± 2	<1	7 ± 3	14 ± 4
YqjM	4 ± 1	2 ± 0	<1	4 ± 1	1 ± 0
OPR3	3 ± 0	4 ± 1	<1	n.d.	n.d.
NerA	10 ± 6	10 ± 5	<1	n.d.	n.d.
*Gk*OYE	10 ± 1	16 ± 2	<1	n.d.	n.d.

a 12-Oxophytodienoate reductase isoenzymes OPR1 and OPR3 (*Lycopersicon esculentum*), YqjM (*Bacillus subtilis*), OYE1 (*Saccharomyces pastorianus*), OYE2 and OYE3 (*Saccharomyces cerevisiae*), nicotinamide-dependent cyclohexenone reductase NCR (*Zymomonas mobilis*), xenobiotic reductases XenA (*Pseudomonas putida*) and XenB (*Pseudomonas fluorescens*), glycerol trinitrate reductase NerA (*Agrobacterium radiobacter*), *Kluyveromyces lactis* yellow enzyme 1 KYE1, *Yersinia bercovieri* ene-reductase YersER, nitroreductase NRSal (*Salmonella typhimurium*), *N*-ethylmaleimide reductase NemR (*Escherichia coli*), pentaerythritol tetranitrate reductase PETNr (*Enterobacter cloacae* PB2), morphinone reductase MR (*P. putida* M10), estrogen-binding protein EBP1 (*Candida albicans*), YcnD and YhdA (*B. subtilis*), Lot6p (*S. cerevisiae*), *Gk*OYE (*Geobacillus kaustophilus* DSM 7263), CrS (*Thermus scotoductus* SA-01); conversions of ≤1% were detected with OPR1, XenB, NemR, PETNr, YcnD, Lot6P, YhdA, KYE1, MR, YersER, and NRSal.

b The reaction was performed according to method A (columns A and B) or method B (columns C and D) using degassed buffer in glass vials which were flushed with argon and sealed with a screw cap lined with a teflon septum. c., conversion; epox, epoxide formed via nonenzymatic Weitz–Scheffer epoxidation; n.d., not determined.

In an attempt to overcome incomplete conversions caused by co-product inhibition exerted by phenol (**1d**), the reaction conditions were optimized in terms of (i) the buffer type and its pH, (ii) the reaction temperature, and (iii) the presence of molecular oxygen.

**Figure 3:**
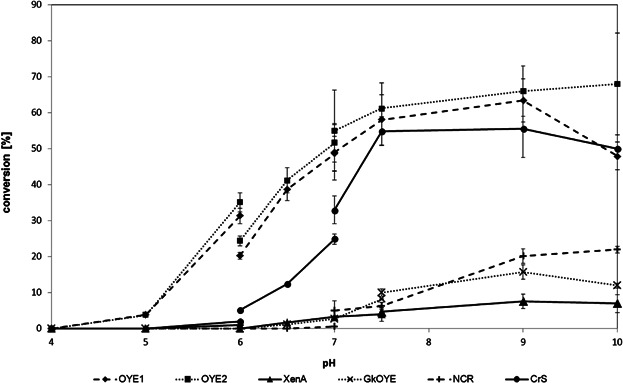
Optimization of buffer-system and pH for the enzymatic disproportionation of cyclohex-2-enone (1a) according to method A. pH 4–6: citrate buffer (50 mM); pH 6–8: phosphate buffer (50 mM); pH 7–10: Tris–HCl buffer (50 mM). Standard conditions: protein content 100 µg/mL; 10 mM 1a; shaking at 120 rpm at 30°C for 24 h.

For the pH-tuning, three different buffer systems (citrate, phosphate, and Tris–HCl) were tested, covering a pH range from 4 to 10 (Fig. 3). Since it was shown that the more electron-rich phenolate-anion dominated over the neutral phenol species in charge-transfer complex formation (Abramovitz and Massey, 1976a; Buckman and Miller, 1998, 2000a, 2000b), elevated pH values are expected to be unfavorable based on the estimated pKa of 7.3 for phenol (**1d**) within the active site of EBP1 (Buckman and Miller, 1998). However, this effect is compensated by destabilization of the charge-transfer complex by action of an acidic amino acid residue in the active site (Tyr206 in EBP1, pKa 9.4) (Buckman and Miller, 1998) acting as proton donor/acceptor on Cα, which is deprotonated under basic conditions, thereby repelling the phenolate species. Overall, the latter effect seemed to dominate because endpoint conversions were enhanced at pH 9 with all enzymes (Fig. 3).

Instead of an enone substrate, ene-reductases are also able to use O_2_ for the oxidation of FMNH_2_ in the oxidative half-reaction. Due to this side-activity, which is related to that of flavin dependent NAD(P)H-oxidases (Hirano et al., 2008; Jiang and Bommarius, 2004; Riebel et al., 2002), H_2_O_2_ is formed, which in turn epoxidizes activated alkenes—such as cyclohex-2-enone (**1a**) (Mueller et al., 2009)—in a subsequent non-enzymatic Weitz–Scheffer epoxidation (Weitz and Scheffer, 1921).

Since this catalytic promiscuity is also supported by high pH, 2,3-epoxycyclohexanone was formed between 0% and 5% at pH 9 (Table 1, column B). In order to suppress the undesired loss of reduction equivalents, anaerobic conditions were applied (Table 1, columns C and D). As expected, the absence of O_2_ completely eliminated the competing epoxidation.

Investigation of the disproportionation rate over a temperature range of 20–70°C revealed typical bell-shaped optima between 40 and 50°C for the mesophilic enzymes, whereas the thermophilic candidates, such as *Gk*OYE and CrS showed the highest conversions at 60 and 50°C, respectively (see Supporting Information). Based on these parameters, all further experiments were performed in Tris–HCl buffer at pH 7.5 and pH 9 under anaerobic conditions at 30°C and 24 h.

Although optimization with respect to pH, temperature, and exclusion of oxygen led to improved disproportionation and suppressed undesired epoxidation (Table 1, columns C and D), the maximum conversions were far from quantitative (*c*_max_ 67% using OYE2), caused by the inhibitory effect of the phenolic co-product. Since the latter is reversible, in situ (co-)product removal (ISPR; Etschmann et al., 2005; Lye and Woodley, 1999; Stark and von Stockar, 2003) of phenol was attempted to raise conversions.

**Figure 4:**
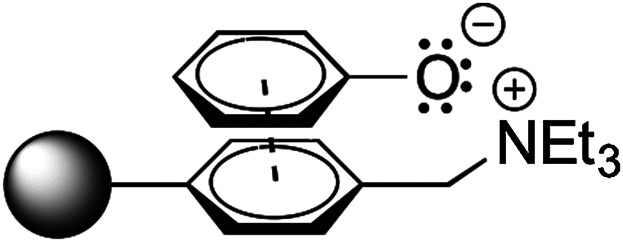
Schematic representation of phenolate binding onto MP-carbonate through π–π stacking and salt bridge.

A search for a suitable phenol-adsorbing polymeric material revealed macroporous polystyrene (MP-)carbonate as a suitable candidate (Lyon and Kercher, 2004; Selwood et al., 2001). The latter possesses positively charged triethylammonium-groups linked to an aromatic styrene moiety, which enables ionic binding of the phenolate anion supported by π–π stacking of both aromatic systems (Fig. 4). The disproportionation of cyclohexenone by OYE1, OYE2, XenA, and CrS was investigated in presence of varying amounts of MP-carbonate. For OYE2 and CrS, a 40-fold loading capacity of adsorbent gave best results by scavenging >90% of phenol, going in hand with considerably enhanced conversions and a near-quantitative value for CrS (c 97%) (Table 2).

**Table 2:** Enzymatic disproportionation of cyclohex-2-enone (1a) in presence of MP-carbonate as phenol scavenger (40-fold loading capacity) according to method C

Enzyme	pH 7.5 c. (%)	pH 9 c. (%)
OYE1	68 ± 9	74 ± 3
XenA	18 ± 4	25 ± 7
OYE2	89 ± 4	90 ± 1
CrS	93 ± 1	97 ± 1

Encouraged by these results, MP-carbonate was used as phenol scavenger in the nicotinamide-independent C=C-bond reduction. For this purpose, two previously employed H-donors—1,4-cyclohexanedione (**5c**) forming 1,4-dihydroxy benzene (**5d**), and 3-methylcyclohex-2-enone (**6c**) forming 3-methylphenol (**6d**) (Stueckler et al., 2010)—were tested with three types of substrates for ene-reductases (Fig. 2).

Overall, the conversions of the substrate-coupled hydrogen transfer system could be considerably improved by the scavenging system. The conversion of the NAD(P)H-free bioreduction of 4-ketoisophorone (**2a**) could be more than doubled from 18% in the absence of scavenger (Table 3, entry 8) to 45% (Table 3, entry 10). In general, 1,4-cyclohexanedione (**5c**) turned out to be a superior H-donor than 3-methylcyclohex-2-enone (**6c**) at basic pH. However, different OYEs preferred different co-substrates: Although XenA gave low conversions in the disproportionation of cyclohex-2-enone (*c*_max_ 25%, Table 2), it exhibited enhanced activities with **5c** and **6c** (*c*_max_ 45% Table 3, entries 10 and 15). In contrast to the majority of enzymes, CrS displayed the highest rates using **6c** as H-donor (*c*_max_ 97% Table 3, entry 17).

**Table 3:** Nicotinamide-independent asymmetric bioreduction of activated alkenes (method C)

Entry	Substrate	Donor	Enzyme	pH	MP-C (eq.)^a^	Conversion (%)	e.e. (%)
1	**2a**	**5c**	OYE1	7.5	0	3 ± 0	74 ± 3 (*R*)
2	**2a**	**5c**	OYE1	9	0	12 ± 5	16 ± 3 (*R*)
3	**2a**	**5c**	XenA	7.5	0	7 ± 1	67 ± 1 (*R*)
4	**2a**	**5c**	XenA	9	0	16 ± 2	10 ± 5 (*R*)
5	**2a**	**6c**	OYE1	7.5	0	2 ± 0	69 ± 2 (*R*)
6	**2a**	**6c**	OYE1	9	0	4 ± 1	21 ± 1 (*R*)
7	**2a**	**6c**	XenA	7.5	0	8 ± 2	66 ± 3 (*R*)
8	**2a**	**6c**	XenA	9	0	18 ± 2	*rac*
9	**2a**	**5c**	OYE1	9	40	66 ± 5	*rac*
10	**2a**	**5c**	XenA	9	40	45 ± 12	*rac*
11	**2a**	**5c**	NerA	9	40	**84** **± 4**	***rac***
12	**2a**	**5c**	*Gk*OYE	9	40	**84** **± 5**	***rac***
13	**2a**	**5c**	CrS	9	40	81 ± 2	*rac*
14	**2a**	**6c**	OYE1	9	40	13 ± 5	*rac*
15	**2a**	**6c**	XenA	9	40	33 ± 12	*rac*
16	**2a**	**6c**	*Gk*OYE	9	40	**77** ± 15	***rac***
17	**2a**	**6c**	CrS	9	40	**97** **± 2**	***rac***
18	**3a**	**5c**	*Gk*OYE	9	40	14 ± 4	>99 ± 0 (*R*)
19	**3a**	**5c**	CrS	9	40	**47** **± 8**	**>99** **± 0 (*R*)**
20	**3a**	**6c**	*Gk*OYE	9	40	7 ± 1	>99 ± 0 (*R*)
21	**3a**	**6c**	CrS	9	40	23 ± 1	>99 ± 0 (*R*)

a Loading capacity of MP-carbonate.

Unfortunately, the parameters leading to optimal conversions—MP-carbonate at elevated pH—caused racemization of (*R*)-levodione [(*R*)-**2b**] (Table 3), which has been observed before (Fryszkowska et al., 2009). However, substrates **3a** and **4a**, leading to stereochemically more stable compounds **3b** and **4b**, were expected to be suitable. In case of **4a**, both, the substrate and the product *N*-phenyl-2-methylsuccinimide (**4b**), showed high affinities to the scavenging resin, which completely inhibited the bioreduction of **4a**. In contrast, dimethyl citraconate (**3a**) was readily reduced by CrS yielding dimethyl (*R*)-2-methylsuccinate [(*R*)-**3b**] in >99% e.e.] at 47% conversion (Table 3, entry 19).

Based on the optimization of the disproportionation of cyclohex-2-enone (**1a**), we finally attempted to further increase the performance of CrS with substrates **2a** and **3a** via reaction engineering. Using the enzyme giving best conversions at given conditions as a starting point, we could push the bioreduction of **2a** to full conversion by raising the enzyme amount, and/or temperature and extending the reaction time, albeit with racemisation of **3a** (Table 4, entries 2–4). In contrast, larger enzyme amounts improved the conversion of **3a** from 47% to 76% (entries 5–7) and by extending the reaction time, a conversion of 92% could be finally reached for (*R*)-**3b** with an e.e. of >99% (entry 8).

**Table 4:** Optimization of NAD(P)H-independent asymmetric bioreduction of 2a and 3a using CrS at pH 9 and MP-carbonate (40 eq. loading capacity) according to method C

Entry	Substrate	Donor	Enzyme amount (µg)	Time (h)	Temp. (°C)	Conversion (%)	e.e. (%)
1	**2a**	**6c**	100	24	30	97 ± 0	*rac*
2	**2a**	**6c**	**200**	24	30	**>99** **± 0**	***rac***
3	**2a**	**6c**	100	**48**	30	**>99** **± 0**	***rac***
4	**2a**	**6c**	100	24	**40**	**>99** **± 0**	***rac***
5	**3a**	**5c**	100	24	30	47 ± 8	>99 **± **0 (*R*)
6	**3a**	**5c**	**200**	24	30	59 ± 2	>99 **± **0 (*R*)
7	**3a**	**5c**	**300**	24	30	76 ± 6	>99 **± **0 (*R*)
8	**3a**	**5c**	**300**	**48**	30	**92** **± 2**	**>99** **± 0 (*R*)**

## Conclusion

From a library of 22 flavin-dependent ene-reductases from the OYE family, 13 candidates were shown to possess strong activities in the NAD(P)H-independent disproportionation of conjugated enones. Limited conversions caused by enzyme inhibition by the co-product phenol forming a charge-transfer complex with the flavin cofactor in the active site could be successfully overcome via ISPR employing MP-carbonate as polymeric phenol-scavenger at elevated pH. Although stereochemically labile compounds, such as α-substituted ketones were incompatible due to racemization, chirally stable α-substituted esters could be obtained for the first time with quantitative conversion via a nicotinamide-independent hydrogen-transfer system.

## Supporting Information

**Figure S1**. Optimization of pH in citrate buffer (50mM) for the disproportionation of cyclohex-2-enone (1a) according to method A. **Figure S2**. Optimization of pH in phosphate buffer (50mM) for the disproportionation of cyclohex-2-enone (1a) according to method A. **Figure S3**. Optimization of pH in Tris-HCl buffer (50mM) for the disproportionation of cyclohex-2-enone (1a) according to method A. **Figure S4**. pH-Dependent epoxide (1e) formation in the disproportionation of cyclohex-2-enone (1a) in Tris-HCl buffer according to method A. **Figure S5**. Temperature profile for the disproportionation of cyclohex-2-enone (1a) according to method A. **Table SI**. Additional data for NAD(P)H-independent bioreduction of alkenes 2a and 3a using H-donors 5c and 6c in presence of MP-carbonate (40 eq. loading capacity) according to method C. n.d., not determined; n.c., no conversion. **Table SII**. Sequence alignment of OYEs from the screening for disproportionation activity with cyclohex-2-enone (1a) (Table I).
