# Supplementary material for: Overcoming co-product inhibition in the nicotinamide independent asymmetric bioreduction of activated C=C-bonds using flavin-dependent ene-reductases
Source: Biotechnol Bioeng. 2013 Jul 10;110(12):3085–92. doi: 10.1002/bit.24981 (PMC4034509; doi:10.1002/bit.24981)
Supplement: Supplementary file 1 — Figure S1. Optimization of pH in citrate buffer (50mM) for the disproportionation of cyclohex-2-enone (1a) according to method A. Figure S2. Optimization of pH in phosphate buffer (50mM) for the disproportionation of cyclohex-2-enone (1a) according to method A. Figure S3. Optimization of pH in Tris-HCl buffer (50mM) for the disproportionation of cyclohex-2-enone (1a) according to method A. Figure S4. pH-Dependent epoxide (1e) formation in the disproportionation of cyclohex-2-enone (1a) in Tris-HCl buffer according to method A. Figure S5. Temperature profile for the disproportionation of cyclohex-2-enone (1a) according to method A. Table SI. Additional data for NAD(P)H-independent bioreduction of alkenes 2a and 3a using H-donors 5c and 6c in presence of MP-carbonate (40 eq. loading capacity) according to method C. n.d., not determined; n.c., no conversion. Table SII. Sequence alignment of OYEs from the screening for disproportionation activity with cyclohex-2-enone (1a) (Table I). [file bit0110-3085-sd1.doc]

**Overcoming Co-Product Inhibition in the Nicotinamide Independent Asymmetric Bioreduction of Activated C=C-Bonds Using Flavin-Dependent Ene-Reductases**

Christoph K. Winkler,^a^ Dorina Clay,^a^ Esta van Heerden,^b^ Kurt Fabera,[[1]](#footnote-2)*

^a^ Department of Chemistry, Organic & Bioorganic Chemistry, University of Graz, Heinrichstrasse 28, A-8010 Graz, Austria;

^b^ Department of Microbial, Biochemical and Food Biotechnology, University of the Free State, 9300 Bloemfontein, South Africa.

**Supporting information**

**Figure S1.** Optimisation of pH in citrate buffer (50mM) for the disproportionation of cyclohex-2-enone (**1a**) according to method A.


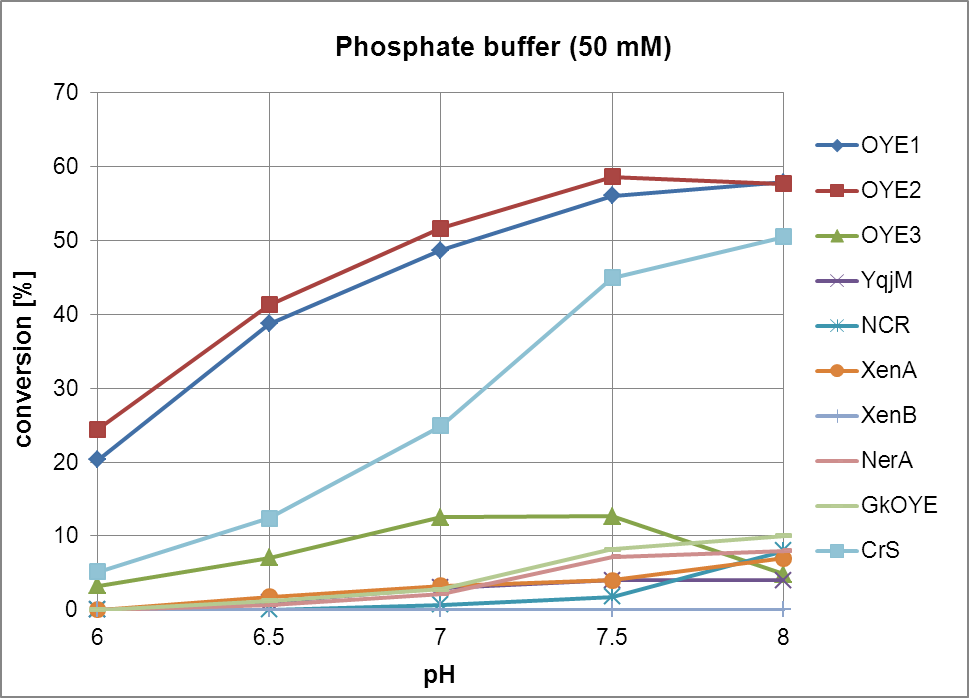


**Figure S2.** Optimisation of pH in phosphate buffer (50mM) for the disproportionation of cyclohex-2-enone (**1a**) according to method A.


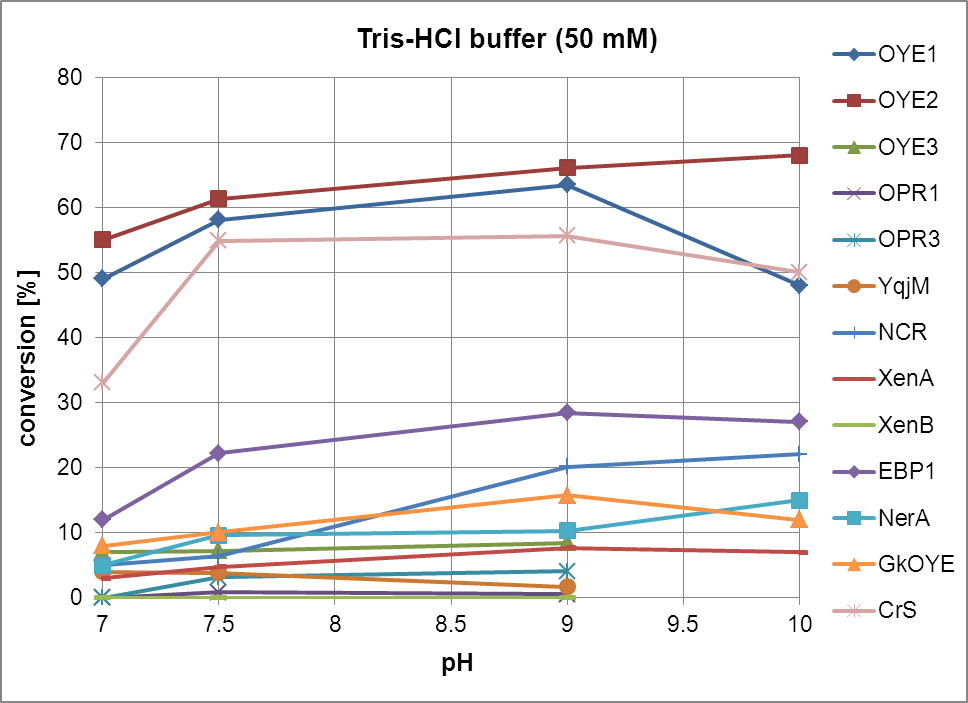


**Figure S3.** Optimisation of pH in Tris-HCl buffer (50mM) for the disproportionation of cyclohex-2-enone (**1a**) according to method A.

**Figure S4.** pH-Dependent epoxide (**1e**) formation in the disproportionation of cyclohex-2-enone (**1a**) in Tris-HCl buffer according to method A.


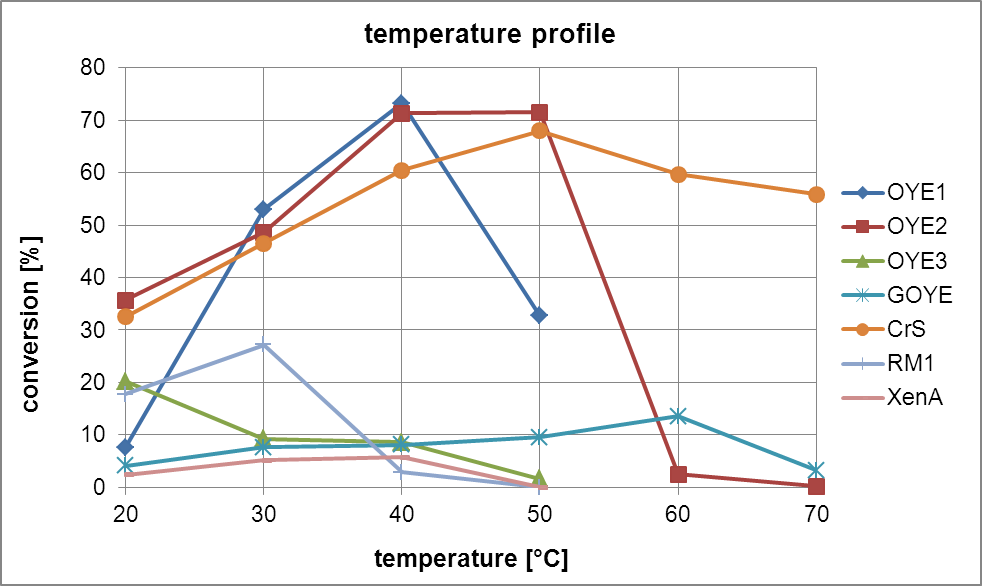


**Figure S5.** Temperature profile for the disproportionation of cyclohex-2-enone (**1a**) according to method A.

**Table S1.** Additional data for NAD(P)H-independent bioreduction of alkenes **2a** and **3a** using H-donors **5c** and **6c** in presence of MP-carbonate (40 eq. loading capacity) according to method C. n.d = not determined; n.c. = no conversion.

| Substrate | Donor | Enzyme | pH | Conversion [%] | e.e. [%] |
| --- | --- | --- | --- | --- | --- |
| **2a** | **5c** | OYE1 | 7.5 | 13 | *rac* |
| **2a** | **5c** | XenA | 7.5 | 20 | *rac* |
| **2a** | **5c** | OYE2 | 7.5 | 26 | *rac* |
| **2a** | **5c** | OYE3 | 7.5 | 14 | *rac* |
| **2a** | **5c** | OPR1 | 7.5 | 5 | *rac* |
| **2a** | **5c** | OPR3 | 7.5 | 9 | *rac* |
| **2a** | **5c** | YqjM | 7.5 | 18 | *rac* |
| **2a** | **5c** | NCR | 7.5 | 40 | *rac* |
| **2a** | **5c** | EBP1 | 7.5 | 14 | *rac* |
| **2a** | **5c** | NerA | 7.5 | 78 | *rac* |
| **2a** | **5c** | *Gk*OYE | 7.5 | 66 | *rac* |
| **2a** | **5c** | CrS | 7.5 | 66 | *rac* |
| **2a** | **5c** | OYE2 | 9 | 51 | *rac* |
| **2a** | **5c** | OYE3 | 9 | 19 | *rac* |
| **2a** | **5c** | OPR1 | 9 | 23 | *rac* |
| **2a** | **5c** | OPR3 | 9 | 23 | *rac* |
| **2a** | **5c** | YqjM | 9 | 13 | *rac* |
| **2a** | **5c** | NCR | 9 | 62 | *rac* |
| **2a** | **5c** | EBP1 | 9 | 24 | *rac* |
| **2a** | **6c** | OYE1 | 7.5 | 3 | *rac* |
| **2a** | **6c** | XenA | 7.5 | 24 | *rac* |
| **2a** | **6c** | OYE2 | 7.5 | 7 | *rac* |
| **2a** | **6c** | OYE3 | 7.5 | 2 | *rac* |
| **2a** | **6c** | OPR1 | 7.5 | 1 | n.d. |
| **2a** | **6c** | OPR3 | 7.5 | 3 | *rac* |
| **2a** | **6c** | YqjM | 7.5 | 10 | *rac* |
| **2a** | **6c** | NCR | 7.5 | 2 | *rac* |
| **2a** | **6c** | EBP1 | 7.5 | 2 | *rac* |
| **2a** | **6c** | NerA | 7.5 | 4 | *rac* |
| **2a** | **6c** | *Gk*OYE | 7.5 | 49 | *rac* |
| **2a** | **6c** | CrS | 7.5 | 85 | *rac* |
| **2a** | **6c** | OYE2 | 9 | 23 | *rac* |
| **2a** | **6c** | OYE3 | 9 | 4 | *rac* |
| **2a** | **6c** | OPR1 | 9 | 3 | *rac* |
| **2a** | **6c** | OPR3 | 9 | 7 | *rac* |
| **2a** | **6c** | YqjM | 9 | 11 | *rac* |
| **2a** | **6c** | NCR | 9 | 3 | *rac* |
| **2a** | **6c** | EBP1 | 9 | 5 | *rac* |
| **2a** | **6c** | NerA | 9 | 5 | *rac* |
| **3a** | **5c** | OYE1 | 7.5 | 2 | >99 (*R*) |
| **3a** | **5c** | OYE2 | 7.5 | 1 | n.d. |
| **3a** | **5c** | YqjM | 7.5 | 1 | n.d. |
| **3a** | **5c** | NCR | 7.5 | 1 | n.d. |
| **3a** | **5c** | XenA | 7.5 | 4 | >99 (*R*) |
| **3a** | **5c** | EBP1 | 7.5 | 3 | >99 (*R*) |
| **3a** | **5c** | NerA | 7.5 | n.c. | n.d. |
| **3a** | **5c** | *Gk*OYE | 7.5 | 10 | >99 (*R*) |
| **3a** | **5c** | CrS | 7.5 | 46 | >99 (*R*) |
| **3a** | **5c** | OYE1 | 9 | 2 | >99 (*R*) |
| **3a** | **5c** | OYE2 | 9 | 2 | >99 (*R*) |
| **3a** | **5c** | YqjM | 9 | 1 | n.d. |
| **3a** | **5c** | NCR | 9 | 1 | n.d. |
| **3a** | **5c** | XenA | 9 | 4 | >99 (*R*) |
| **3a** | **5c** | EBP1 | 9 | 4 | >99 (*R*) |
| **3a** | **5c** | NerA | 9 | n.c. | n.d. |
| **3a** | **6c** | OYE1 | 7.5 | n.c. | n.d. |
| **3a** | **6c** | OYE2 | 7.5 | n.c. | n.d. |
| **3a** | **6c** | YqjM | 7.5 | 3 | >99 (*R*) |
| **3a** | **6c** | NCR | 7.5 | n.c. | n.d. |
| **3a** | **6c** | XenA | 7.5 | 15 | >99 (*R*) |
| **3a** | **6c** | EBP1 | 7.5 | <1 | n.d. |
| **3a** | **6c** | NerA | 7.5 | n.c. | n.d. |
| **3a** | **6c** | *Gk*OYE | 7.5 | 7 | >99 (*R*) |
| **3a** | **6c** | CrS | 7.5 | 14 | >99 (*R*) |
| **3a** | **6c** | OYE1 | 9 | n.c. | n.d. |
| **3a** | **6c** | OYE2 | 9 | n.c. | n.d. |
| **3a** | **6c** | YqjM | 9 | 1 | n.d. |
| **3a** | **6c** | NCR | 9 | n.c. | n.d. |
| **3a** | **6c** | XenA | 9 | 9 | >99 (*R*) |
| **3a** | **6c** | EBP1 | 9 | <1 | n.d. |
| **3a** | **6c** | NerA | 9 | n.c. | n.d. |

**Table S2. Sequence alignment of OYEs from the screening for disproportionation activity with cyclohex-2-enone (1a) (Table I).**

* 20 * 40 * 60 * 80 * 100
OYE1 : -----------MSFVKDFKPQALGDTNLFKPIKIGNNELLHRAVIPPLTRMRALHPGNIPNRDWAVEYYTQRAQR--------PGTMIITEGAFISPQAG : 81
OYE2 : -----------MPFVKDFKPQALGDTNLFKPIKIGNNELLHRAVIPPLTRMRAQHPGNIPNRDWAVEYYAQRAQR--------PGTLIITEGTFPSPQSG : 81
EBP1 : MTIESTNSFVVPSDTKLIDVTPLGSTKLFQPIKVGNNVLPQRIAYVPTTRFRASKDH--IPSDLQLNYYNARSQY--------PGTLIITEATFASERGG : 90
NCR : ------------------------MPSLFDPIRFGAFTAKNRIWMAPLTRGRA-TRDHVPTE-IMAEYYAQRASA----------GLIISEATGISQEGL : 64
TS : ------------------------MALLFTPLELGGLRLKNRLAMSPMCQYSAT-LEGEVTD-WHLLHYPTRALG--------GVGLILVEATAVEPLGR : 66
OYE3 : -----------MPFVKGFEPISLRDTNLFEPIKIGNTQLAHRAVMPPLTRMRATHPGNIPNKEWAAVYYGQRAQR--------PGTMIITEGTFISPQAG : 81
LeOPR3 : ----------------MASSAQDGNNPLFSPYKMGKFNLSHRVVLAPMTRCRA--LNNIPQA-ALGEYYEQRATA---------GGFLITEGTMISPTSA : 72
YqjM : -----------------------MARKLFTPITIKDMTLKNRIVMSPMCMYSSHEKDGKLTP-FHMAHYISRAIG--------QVGLIIVEASAVNPQGR : 68
NerA : ------------------------MTSLFEPAQAGDIALANRIVMAPLTRNRS--PGAIPNN-LNATYYEQRATA----------GLIVTEGTPISQQGQ : 63
XenA : ------------------------MSALFEPYTLKDVTLRNRIAIPPMCQYMA--EDGLIND-WHQVHYASMARG--------GAGLLVVEATAVAPEGR : 65
GkOYE : -----------------------MNTMLFSPYTIRGLTLKNRIVMSPMCMYSCDTKDGAVRT-WHKIHYPARAVG--------QVGLIIVEATGVTPQGR : 68
KYE : -----------MSF-MNFEPKPLADTDIFKPIKIGNTELKHRVVMPALTRMRALHPGNVPNPDWAVEYYRQRSQY--------PGTMIITEGAFPSAQSG : 80
LeOPR1 : ------------MENKVVEEKQVDKIPLMSPCKMGKFELCHRVVLAPLTRQRS--YGYIPQP-HAILHYSQRSTN---------GGLLIGEATVISETGI : 76
MR : -----------------MPDTSFSNPGLFTPLQLGSLSLPNRVIMAPLTRSR--TPDSVPGR-LQQIYYGQRASA----------GLIISEATNISPTAR : 70
PETNred : ----------------------MSAEKLFTPLKVGAVTAPNRVFMAPLTRLRSIEPGDIPTP-LMGEYYRQRASA----------GLIISEATQISAQAK : 67
XenB : ------------------------MATIFDPIKLGDLELSNRIIMAPLTRCRA-DEGRVPNA-LMAEYYVQRASA----------GLILSEATSVTPMGV : 64
YersER : -------------------------------MKVGALTLPNRVFMAPLTRLRSIEPGDIPTP-LMAEYYRQRASA----------GLIITEATQISFQAK : 58
Lot6p : ------------------------MKV---GIIMGSVRAKRVCPEIAAYVKRTIE-----N---SEELIDQKLKIQVVDLQQIALPLYEDDDELIPA--- : 62
YhdA : ------------------------MNM---LVINGTPRKHGRTRIA-------------------ASYIAALYHTDLIDLSEFVLPVFNGEAEQSE---- : 50
Nem-R : ----------------------MSSEKLYSPLKVGAITAANRIFMAPLTRLRSIEPGDIPTP-LMAEYYRQRASA----------GLIISEATQISAQAK : 67
Ycnd : ----------------------------------------MNEVIKSL-----------------TDHRSIRS--------------------------- : 16
NRSal : ----------------------------------------MDIVSVAL-----------------Q-RYSTKA--------------------------- : 15
 r y r e

* 120 * 140 * 160 * 180 * 200
OYE1 : GYDNAPGVWSEEQMVEWTKIFNAIHEKKSFVWVQLWVLGWAAFPDNLAR----------DGLRYDSASDNV------FMDAEQEAKAKKANNPQHSLTKD : 165
OYE2 : GYDNAPGIWSEEQIKEWTKIFKAIHENKSFAWVQLWVLGWAAFPDTLAR----------DGLRYDSASDNV------YMNAEQEEKAKKANNPQHSITKD : 165
EBP1 : IDLHVPGIYNDAQAKSWKKINEAIHGNGSFSSVQLWYLGRVANAKDLKD----------SGLPLIAP-SAV------YWDENSEKLAKEAGNELRALTEE : 173
NCR : GWPYAPGIWSDAQVEAWLPITQAVHDAGGLIFAQLWHMGRMVPSN--VS-----------GMQPVAPSASQAPGLGHT------YDGKKPYDVARALRLD : 145
TS : ISPYDLGIWSEDHLPGLKELARRIREAGAVPGIQLAHAGRKAGTARPWEGGKP------LGWRVVGPSPIPF--------------DEGYP-VPEPLDEA : 145
OYE3 : GYDNAPGIWSDEQVAEWKNIFLAIHDCQSFAWVQLWSLGWASFPDVLAR----------DGLRYDCASDRV------YMNATLQEKAKDANNLEHSLTKD : 165
LeOPR3 : GFPHVPGIFTKEQVREWKKIVDVVHAKGAVIFCQLWHVGRASHEVYQPA-----------GAAPISSTEKPISNR---WRILMPDGTHGIYPKPRAIGTY : 158
YqjM : ITDQDLGIWSDEHIEGFAKLTEQVKEQGSKIGIQLAHAGRKAELE----------------GDIFAPSAIAF--------------DEQSA-TPVEMSAE : 137
NerA : GYADVPGLYKREAIEGWKKITDGVHSAGGKIVAQIWHVGRISHTSLQPH-----------GGQPVAPSAITAKSKT-YIINDDGTGAFAETSEPRALTID : 151
XenA : ITPGCAGIWSDAHAQAFVPVVQAIKAAGSVPGIQIAHAGRKASANRPWEGDDHIGADDARGWETIAPSAIAF--------------GAHLPNVPRAMTLD : 151
GkOYE : ISERDLGIWSDDHIAGLRELVGLVKEHGAAIGIQLAHAGRKSQVP----------------GEIIAPSAVPF--------------DDSSP-TPKEMTKA : 137
KYE : GYDNAPGVWSEEQLAQWRKIFKAIHDNKSFVWVQLWVLGRQAFADNLAR----------DGLRYDSASDEV------YMGEDEKERAIRSNNPQHGITKD : 164
LeOPR1 : GYKDVPGIWTKEQVEAWKPIVDAVHAKGGIFFCQIWHVGRVSNKDFQPN-----------GEDPISCTDRGLTPQ---IRSNGI--DIAHFTRPRRLTTD : 160
MR : GYVYTPGIWTDAQEAGWKGVVEAVHAKGGRIALQLWHVGRVSHELVQPD-----------GQQPVAPSALKAEGAECFVEFEDGTAGLHPTSTPRALETD : 159
PETNred : GYAGAPGLHSPEQIAAWKKITAGVHAEDGRIAVQLWHTGRISHSSIQPG-----------GQAPVSASALNANTRT-SLRDENGNAIRVDTTTPRALELD : 155
XenB : GYPDTPGIWSNDQVRGWTNITKAVHAAGGKIVLQLWHVGRISHPL-YLN-----------GEAPVAPSAIQPKGHV-SLVR-----PLADYPTPRALETA : 146
YersER : GYAGAPGLHTQEQLNAWKKITQAVHEEGGHIAVQLWHVGRISHSSLQPG-----------QQAPVAPSAIAADTRT-TVRDENGAWVRVPCSTPRALETE : 146
Lot6p : -QIKSVDEYADSKTRSWSRIVNALDI-------------------------------------------------------------------------- : 87
YhdA : ----------LLKVQELKQRVTKADA-------------------------------------------------------------------------- : 66
Nem-R : GYAGAPGIHSPEQIAAWKKITAGVHAENGHMAVQLWHTGRISHASLQPG-----------GQAPVAPSALSAGTRT-SLRDENGQAIRVETSMPRALELE : 155
Ycnd : -----Y-TDEPVAQEQLDQIIEAVQSAPSSINGQQVTVITVQDKERKK---------------KISELAGGQ-----PWI-------------------D : 71
NRSal : -----FDPSKKLTAEEADKIKTLLQYSPSSTNSQPWHFIVASTEEGKA---------------RVAKSAAGN-----YTFNERKM--------------L : 76
 g q g

* 220 * 240 * 260 * 280 * 300
OYE1 : EIKQYI-KEYVQAAKNSIAAGADGVEIHSANGYLLNQFLDPHSNTRTDEYG-GSIENRARFTLEVVDALVE---------------AIGHE---KVGLRL : 245
OYE2 : EIKQYV-KEYVQAAKNSIAAGADGVEIHSANGYLLNQFLDPHSNNRTDEYG-GSIENRARFTLEVVDAVVD---------------AIGPE---KVGLRL : 245
EBP1 : EIDHIVEVEYPNAAKHALEAGFDYVEIHGAHGYLLDQFLNLASNKRTDKYGCGSIENRARLLLRVVDKLIE---------------VVGAN---RLALRL : 255
NCR : EIPRLL-DDYEKAARHALKAGFDGVQIHAANGYLIDEFIRDSTNHRHDEYG-GAVENRIRLLKDVTERVIA---------------TIGKE---RTAVRL : 225
TS : GMERIL-QAFVEGARRALRAGFQVIELHMAHGYLLSSFLSPLSNQRTDAYG-GSLENRMRFPLQVAQAVRE---------------VVPRELPLFVRVSA : 228
OYE3 : DIKQYI-KDYIHAAKNSIAAGADGVEIHSANGYLLNQFLDPHSNKRTDEYG-GTIENRARFTLEVVDALIE---------------TIGPE---RVGLRL : 245
LeOPR3 : EISQVV-EDYRRSALNAIEAGFDGIEIHGAHGYLIDQFLKDGINDRTDEYG-GSLANRCKFITQVVQAVVS---------------AIGAD---RVGVRV : 238
YqjM : KVKETV-QEFKQAAARAKEAGFDVIEIHAAHGYLIHEFLSPLSNHRTDEYG-GSPENRYRFLREIIDEVKQ---------------VW--DGPLFVRVSA : 218
NerA : DIGLIL-EDYRSGARAALEAGFDGVEIHAANGYLIEQFLKSSTNQRTDDYG-GSIENRARFLLEVVDAVAE---------------EIGAG---RTGIRL : 231
XenA : DIARVK-QDFVDAARRARDAGFEWIELHFAHGYLGQSFFSEHSNKRTDAYG-GSFDNRSRFLLETLAAVRE---------------VWPENLPLTARFGV : 234
GkOYE : DIEETV-QAFQNGARRAKEAGFDVIEIHAAHGYLINEFLSPLSNRRQDEYG-GSPENRYRFLGEVIDAVRE---------------VW--DGPLFVRISA : 218
KYE : EIKQYI-RDYVDAAKKCIDAGADGVEIHSANGYLLNQFLDPISNKRTDEYG-GSIENRARFVLEVVDAVVD---------------AVGAE---RTSIRF : 244
LeOPR1 : EIPQIV-NEFRVAARNAIEAGFDGVEIHGAHGYLIDQFMKDQVNDRSDKYG-GSLENRCRFALEIVEAVAN---------------EIGSD---RVGIRI : 240
MR : EIPGIV-EDYRQAAQRAKRAGFDMVEVHAANACLPNQFLATGTNRRTDQYG-GSIENRARFPLEVVDAVAE---------------VFGPE---RVGIRL : 239
PETNred : EIPGIV-NDFRQAVANAREAGFDLVELHSAHGYLLHQFLSPSSNQRTDQYG-GSVENRARLVLEVVDAVCN---------------EWSAD---RIGIRV : 235
XenB : EIAEIV-EAYRTGAENAKAAGFDGVEIHGANGYLLDQFLQSSTNQRTDNYG-GSLENRARLLLEVTDAAID---------------VWGAG---RVGVHL : 226
YersER : EIPGII-NDFRQATANAREAGFDYIELHAAHGYLLHQFMSPASNQRTDQYG-GSIENRTRLTLEVVDATAA---------------QWSAE---RIGIRI : 226
Lot6p : --IVFVTPQYNWGYPAALKNAID---------RLY----------------------------------------------------------------- : 111
YhdA : --IVLLSPEYHSGMSGALKNALD---------FLS----------------------------------------------------------------- : 90
Nem-R : EIPGIV-NDFRQAIANAREAGFDLVELHSAHGYLLHQFLSPSSNHRTDQYG-GSVENRARLVLEVVDAGIE---------------EWGAD---RIGIRV : 235
Ycnd : QAPVFL--LFCADFNRAKIALEDL----------------------------------HDFKMEITNGLESVLVGAVDAGIALGTATAAAE---SLGLGT : 132
NRSal : DASHVV--VFCAKTAMDDAWLERVVDQEDADGRFA----TPEAKAAND-KGRRFFADMHRVSLKDDHQWM-----AKQVYLNVGNFLLGVA---AMGLDA : 161
 ag d h a g l f n r d yg g nr r

* 320 * 340 * 360 * 380 * 400
OYE1 : SPYGVFNSMSGGAETGIVAQYAYVAGELEKRA-KAGKRL-AFVHLVEPRVTNPFLTEGEG----EYEGGSNDFVYSIWK---GPVIRAGN---------- : 326
OYE2 : SPYGVFNSMSGGAETGIVAQYAYVLGELERRA-KAGKRL-AFVHLVEPRVTNPFLTEGEG----EYNGGSNKFAYSIWK---GPIIRAGN---------- : 326
EBP1 : SPWASFQGMEIEGE----EIHSYILQQLQQRA-DNGQQL-AYISLVEPRVTGIYDVSLK-----DQQGRSNEFAYKIWK---GNFIRAGN---------- : 331
NCR : SPNGEIQGTVDS-HPE--QVFIPAAKMLSDLD------I-AFLGMREGAVDGTFGKTDQPKL-------S-PEIRKVFK---PPLVLNQD---------- : 294
TS : TDWGE-GGWSL-------EDTLAFARRLKELG------V-DLLDCSSGGVVLRVRIPLAP----GFQVPFADAVRKRVG---LRTGAVGL---------- : 296
OYE3 : SPYGTFNSMSGGAEPGIIAQYSYVLGELEKRA-KAGKRL-AFVHLVEPRVTDPSLVEGEG----EYSEGTNDFAYSIWK---GPIIRAGN---------- : 326
LeOPR3 : SPAIDHLDAMDS-NPL--SLGLAVVERLNKIQLHSGSKL-AYLHVTQPRYVAYGQTEAGRLGSEEEEARLMRTLRNAYQ---GTFICSGG---------- : 321
YqjM : SDYTD-KGLDI-------ADHIGFAKWMKEQG------V-DLIDCSSGALV-HADINVFP----GYQVSFAEKIREQAD---MATGAVGM---------- : 285
NerA : SPVTPANDIFEA-DPQ--PLYNYVVEQLGKRN------L-AFIHVVEGATGGPRDFKQGDKPF-DYASFKAAYRNAGGK---GLWIANNG---------- : 307
XenA : LEYDGRDEQTL-------EESIELARRFKAGG------L-DLLSVSVGFTIPETNIPWGP----AFMGPIAERVRREAK---LPVTSAWG---------- : 303
GkOYE : SDYHP-DGLTA-------KDYVPYAKRMKEQG------V-DLVDVSSGAIV-PARMNVYP----GYQVPFAELIRREAD---IPTGAVGL---------- : 285
KYE : SPYGVFGTMSGGSDPVLVAQFAYVLAELEKRA-KAGKRL-AYVDLVEPRVTSPFQPEFEG----WYKGGTNEFVYSVWK---GNVLRVGN---------- : 325
LeOPR1 : SPFAHYNEAGDT-NPT--ALGLYMVESLNKYD------L-AYCHVVEPRMKTAWE--------KIECTESLVPMRKAYK---GTFIVAGG---------- : 309
MR : TPFLELFGLTDD-EPE--AMAFYLAGELDRRG------L-AYLHFNEPDWIG------GDITY-PE-GFR-EQMRQRFK---GGLIYCGN---------- : 307
PETNred : SPIGTFQNVDNGPNEE--ADALYLIEELAKRG------I-AYLHMSETDLAG------GK-PY-SE-AFR-QKVRERFH---GVIIGAGA---------- : 303
XenB : APRADSHDMGDD-NLA--ETFTYVARELGKRG------I-AFICSREKEGADSLG------P---------Q-LKEAFG---GAYIANER---------- : 287
YersER : SPLGPFNGLDNGEDQE--EAALYLIDELNKRH------I-AYLHISEPDWAG------GK-PY-SE-AFR-DAVRARFK---GVIIGAGA---------- : 294
Lot6p : ------------------------HEW---HG------K-PALVVSYGGHGGS------------K---CNDQLQEVLHGLKMNVIGGVAVKIPVGT--- : 159
YhdA : ------------------------SEQFKYKP------V-ALLAVAGGGKGGI------------N---ALNNMRTVMRGVYANVIPKQLVLDPVHIDVE : 144
Nem-R : SPIGTFQNTDNGPNEE--ADALYLIEQLGKRG------I-AYLHMSEPDWAG------GE-PY-TD-AFR-EKVRARFH---GPIIGAGA---------- : 303
Ycnd : VPIGAVRGNPQEL--------------------IELLELPKYVFPLSGLVIGHPA------------------DRSAKK-----------PRLPQEAVNH : 183
NRSal : VPIEGFDAEVLDAEFG-----------LKEKGYTSLVVVPVGHHSVEDFNAGLP------------------------K-----------SRLPLETTLT : 215

* 420 * 440 * 460 * 480
OYE1 : -FAL-HPE---VVREEVKDK-R-TLIGYGRFFISNPDLVDRLEKGLPLNKYD--R--DTFYQMSA-HGYIDYPTYEEALKLGWDKK-- : 400
OYE2 : -FAL-HPE---VVREEVKDP-R-TLIGYGRFFISNPDLVDRLEKGLPLNKYD--R--DTFYKMSA-EGYIDYPTYEEALKLGWDKN-- : 400
EBP1 : -YTY-DAPEFKTLINDLKND-R-SIIGFSRFFTSNPDLVEKLKLGKPLNYYN--R--EEFYKYYN-YGYNSYDESEKQVIGKPLA--- : 407
NCR : -YTF-ETAQA----ALDSGV-A-DAISFGRPFIGNPDLPRRFFEKAPLTKDV--I--ETWYTQTP-KGYTDYPLLGD----------- : 358
TS : -ITTPEQAET----LLQAGS-A-DLVLLGRVLLRDPYFPLRAAKALGVAPEV-----PPQYQR----GF------------------- : 349
OYE3 : -YAL-HPE---VVREQVKDP-R-TLIGYGRFFISNPDLVYRLEEGLPLNKYD--R--STFYTMSA-EGYTDYPTYEEAVDLGWNKN-- : 400
LeOPR3 : -YTR-ELGIE----AVAQGD-A-DLVSYGRLFISNPDLVMRIKLNAPLNKYN--R--KTFYTQDPVVGYTDYPFLQGNGSNGPLSRL- : 396
YqjM : -ITDGSMAEE----ILQNGR-A-DLIFIGRELLRDPFFARTAAKQLNTEIPA-----PVQYER----GW------------------- : 338
NerA : -YDR-QSAIE----AVESGK-V-DAVAFGKAFIANPDLVRRLKNDAPLNAPN--Q--PTFYGGGA-EGYTDYPALAQ----------- : 371
XenA : -FGTPQLAEA----ALQANQ-L-DLVSVGRAHLADPHWAYFAAKELGVEKASWTL--PAPYAHWL-ERYR------------------ : 363
GkOYE : -ITSGWQAEE----ILQNGR-A-DLVFLGRELLRNPYWPYAAARELGAKISA-----PVQYER----GWRF----------------- : 340
KYE : -YAL-DPD---AAITDSKNP-N-TLIGYGRAFIANPDLVERLEKGLPLNQYD--R--PSFYKMSA-EGYIDYPTYEEAVAKGYKK--- : 398
LeOPR1 : -YDR-EDGNR----ALIEDR-A-DLVAYGRLFISNPDLPKRFELNAPLNKYN--R--DTFYTSDPIVGYTDYPFLETMT--------- : 376
MR : -YDA-GRAQA----RLDDNT-A-DAVAFGRPFIANPDLPERFRLGAALNEPD--P--STFYGGAE-VGYTDYPFLDNGHDRLG----- : 377
PETNred : -YTA-EKAED----LIGKGL-I-DAVAFGRDYIANPDLVARLQKKAELNPQR--P--ESFYGGGA-EGYTDYPSL------------- : 365
XenB : -FTK-DSANA----WLAEGK-A-DAVAFGVPFIANPDLPARLKADAPLNEPR--P--ELFYGKGP-VGYIDYPTL------------- : 349
YersER : -YTA-EKAEE----LIEKGF-I-DAVAFGRSYISNPDLVARLQQHAPLNEPD--G--ETFYGGGA-KGYTDYPTL------------- : 356
Lot6p : -IPLPEDIVP-QLSVHNEEI-L-QLLASCIETTRNK---------------------------------------------------- : 191
YhdA : NATVAENIKE-SIK----EL-V-EELSM-FAKAGNPGV-------------------------------------------------- : 174
Nem-R : -YTV-EKAET----LIGKGL-I-DAVAFGRDWIANPDLVARLQRKAELNPQR--A--ESFYGGGA-EGYTDYPTL------------- : 365
Ycnd : QET-----------YLNQDELTSHIQAYDEQM-----SEYMNKRTNGKETRNWSQSIASYYER------LYYPHIREMLEKQGFKVEK : 249
NRSal : EV-------------------------------------------------------------------------------------- : 217
 p y

1. ^*^ Corresponding author: <Kurt.Faber@Uni-Graz.at>, phone+43-316-380-5332. [↑](#footnote-ref-2)
